# Native *Bacillus paralicheniformis* isolate as a potential agent for phytopathogenic nematodes control

**DOI:** 10.3389/fmicb.2023.1213306

**Published:** 2023-07-31

**Authors:** Estefany Chavarria-Quicaño, Victor Contreras-Jácquez, Armando Carrillo-Fasio, Francisco De la Torre-González, Ali Asaff-Torres

**Affiliations:** ^1^Laboratory of Industrial Biotechnology, Department of Food Science, Centro de Investigación en Alimentación y Desarrollo, Hermosillo, Mexico; ^2^Laboratory of Nematology, Centro de Investigación en Alimentación y Desarrollo, Culiacán, Mexico; ^3^Innovak Global, Chihuahua, Mexico

**Keywords:** bacterial screening, biocontrol, nematicidal secretomes, bionematicides, *Bacillus*

## Abstract

Phytopathogenic nematodes (PPNs) are responsible for substantial damages within agricultural crops worldwide which can be controlled employing beneficial microorganisms and/or their metabolites in an ecofriendly way. Nevertheless, the success of the control regards not only on the virulence of the strains or the toxicity of their metabolites but also on their ability to colonize and remain in the rhizospheric environment, particularly in those crops affected by abiotic stresses promoted by the climate change. Consequently, the bioprospection of beneficial microorganisms able to control PPNs and to thrive in adverse conditions has attracted attention. On this way, deserts are perfect ecological niches to isolate microorganisms adapted to harsh enviroments. The purpose of this research was to isolate and characterize bacteria from rhizospheric soil samples collected in the Northwestern Desert of Mexico with potential for PPNs control. As first screening, secretomes of each isolate were tested *in vitro* for nematicidal activity (NA). Then, activities from secretomes and endospores from the selected isolate were confirmed *in vivo* assays. From 100 thermotolerant isolates, the secretome of the isolate identified as *Bacillus paralicheniformis* TB197 showed the highest NA (>95%) against *Meloidogyne incognita*, both *in vitro* and *in vivo* tests, suppressing infections caused by *M. enterolobii* in tomato crops, too. In open field tests, the endospores of TB197 strain showed a reduction of 81% in the infection severity caused by *M. enterolobii* (*p* ≤ 0.05), while the galling index (GI) was reduced 84% (*p* ≤ 0.05) in tomato greenhouse-tests. Also, a reduction of the root necrosis (81%) caused by *Radopholus similis* in banana plantations (*p* ≤ 0.05), compared to the control was observed. Owing to their efficacy in controlling PPNs infections, the endospores and secondary metabolites of *B. paralicheniformis* TB197 strain could be used in bionematicidal formulations.

## 1. Introduction

Phytopathogenic nematodes (PPNs) are one of the greatest threats to agricultural, ornamental and forestry activities (Archidona-Yuste et al., 2019). According to the report of the American Society of Phytopathology (APS), losses in agricultural production caused by PPNs are approximately 14%, which translates into almost 125 billion dollars annually (Mesa-Valle et al., 2020). A common feature of PPNs is the stylet located in the stoma or in the mouth that allows them to cross the host cell wall and inject enzymes that partially digest the cellular content before the nematode sucks it into its digestive system (Kimenju et al., 2009). According to their life cycle and feeding behavior, PPNs are classified into three groups: endoparasites, ectoparasites and semiendoparasites (Gullino et al., 2020). Endoparasites penetrate inside the plant, where all or part of their life cycle occurs; they are mainly root-knot nematodes (e.g., *Meloidogyne* spp.), encysted nematodes (e.g., *Heterodera* spp., *Globodera* spp.), and root-injuring nematodes (e.g., *Pratylenchus* spp., *Hirschmanniella* spp., *Radopholus* spp.), among others (Pires et al., 2022). In contrast, the ectoparasite life cycle occurs entirely outside the plant, piercing the epidermis or the superficial layers of the root cortex of the host plant with the stylet (e.g., *Xiphinema* spp., *Trichodorus* spp., *Paratrichodorus* spp.); therefore, they can also be found in seeds, stems and the aerial part of the plant (leaves and flowers; Pires et al., 2022). Finally, semiendoparasites can partially penetrate the plant, leaving part of the nematode inside the plant and another part outside, laying eggs toward the outside (e.g., *Sphaeronema* spp., *Hoplolaimus* spp., and *Helycotylenchus* spp.; Mesa-Valle et al., 2020).

Any agricultural crop can be affected by the action of some PPNs, and their effect is often underestimated as they produce nonspecific symptoms that are often confused with situations of water stress, nutritional disorders, soil fertility problems, or other secondary fungal or bacterial infections (Pires et al., 2022). Additionally, PPNs have fast generation times, high reproductive rates, and cryptic behaviors, contributing to the difficulties experienced in effectively controlling PPNs infections (Naz and Khan, 2013; Horak et al., 2019). Several strategies have been reported for the control of PPNs infections, such as nonhost crop rotation, soil solarization and the use of chemical nematicides, which include halogenated aliphatic hydrocarbons (e.g., 1,3-dichloropropene), methyl isothiocyanate, oxamyl, thionazine, and carbofuran (Engelbrecht et al., 2018; Naz et al., 2021). Although the use of chemical nematicides is the most successful strategy for the control of PPNs, their long-term repeated use leads to increased nematode resistance, exacerbating the difficulty of nematode management (Chen et al., 2020). In addition, there is evidence of their negative impact on the environment, for example, the groundwater contamination, disturbance of soil fertility, and the accumulation of chemical residues within the trophic levels of soil ecosystems (Engelbrecht et al., 2018). As eco-friendly alternatives, the use of microorganisms and/or their metabolites as biological control agents has attracted scientific interest due to their minimal environmental side effects (He et al., 2020).

Previous studies have shown that certain plant-associated rhizosphere microorganisms belonging to the *Pasteuria*, *Pseudomonas*, *Streptomyces*, and *Bacillus* genera control and antagonize PPNs through their ability to colonize the rhizosphere and secrete enzymes and/or secondary metabolites that are toxic to PPNs and/or elicit an induced systemic response (ISR) in plants (Berini et al., 2018; Zhao et al., 2018; Horak et al., 2019). However, in addition to their nematode control capabilities, this kind of microorganism must thrive under adverse conditions such as droughts, high temperatures and/or soil salinity promoted by climate change, which has been dramatically accelerated in recent years (Bokhari et al., 2020). Indeed, recent research has focused on the bioprospecting of plant-associated beneficial microorganisms capable of adapting to adverse conditions (Engelbrecht et al., 2018; Bokhari et al., 2020).

Desert soils are characterized by extreme conditions, such as high temperatures, water scarcity, high soil salinity, low nutrient levels and high UV radiation (Alsharif et al., 2020; Franklin et al., 2021). In addition, the survival of plants in desert areas is intrinsically related to their microbial communities (Sayed et al., 2019; Bokhari et al., 2020). Alsharif et al. (2020), Bokhari et al. (2020), and Franklin et al. (2021) showed that desert microorganisms not only relieve water stress in plants and promote growth but also protect the plant against pathogens, such as PPNs. Moreover, Viljoen and Labuschagne (2019) and Abd-Elgawad and Mahfouz (2020) found that *Streptomyces* and *Bacillus* strains isolated from the Egyptian desert are capable of effectively controlling PPNs and surviving in extreme conditions of drought and water stress. In this way, the plant-associated microbiota from deserts is a perfect ecological niche to isolate new microorganisms that are well adapted to extreme conditions and have potential for the biological control of phytopathogens, including PPNs.

The Sonoran Desert is one of the warmest regions in North America and in the world, where temperatures above 50°C have been recorded and cover a territory of 260,000 km^2^, including southern Arizona and southeastern California, EUA; northeast, north and south-central Baja California; and north-central and western Sonora, Mexico (Zolotokrylin et al., 2020; Flower et al., 2021). However, unlike other deserts, the Sonoran Desert is the most biodiverse; this is the case for flora, with approximately 2,500 species identified (Andrew et al., 2012; del Conagua, 2020; Franklin et al., 2021). This is relevant under the premise that the microbial communities are important for the survival of these plants, constituting a diverse and untapped pool of microorganisms with the potential to control phytopathogenic agents, such as PPNs, and to support extreme biotic and abiotic conditions or to synthesize thermostable or operatively stable metabolites. For example, Galaviz et al. (2018) and Bashan et al. (2012) found that the Sonoran Desert is home to plant growth-promoting rhizobacteria (PGPRs), such as *Azospirillum brasilense* and *Bacillus pumilus*. However, research on the potential of microorganisms isolated from this ecological niche for biological control of PPNs is scarce. Hence, the objectives of this research were to isolate, characterize and apply bacteria from the rhizosphere of plants growing in the Sonoran Desert with the potential for PPNs control.

## 2. Materials and methods

### 2.1. Reagents

Malt extract, yeast extract, LB broth and bacteriological agar were purchased from BD Difco (Sparks, MD, United States). Methanol and chloroform were provided by J.T. Baker (Xalostoc, Edo. Mex., Mexico). Glucose was provided by Faga Lab (Guamúchil, Sin., México). Verango^®^ [fluopyram, 41.7% (w/v)], Lila Plus^®^ [*Paecilomyces lilacinus* 3% (w/w)] and Nemacem^®^ [Aqueous extract of *Tagetes erecta*, Alpha terthienyl 10% (w/w)] were provided by a local supplier.

### 2.2. Rhizospheric soil sample collection from the Sonoran Desert

Three zones of the Sonoran Desert were chosen for soil sampling: The Pinacate Biosphere Reserve and the Great Desert Altar (latitude: 31°31′55.01′′N, longitude: 113°25′40.05′′ W), La Primavera (latitude: 28°48′09.70′′ N, longitude: 111°12′13.20′′ W) and El Apache (28°18′59.60′′ N, 111°14′40.60′′ W). Two kilograms of rhizospheric soil were collected from at least 10 randomly selected points within each zone. The samples were collected with a small shovel from depths 0–30 cm, immediately placed in sterile plastic bags, refrigerated, and transported to the lab for processing on the same day.

### 2.3. Isolation of bacteria

Ten grams of soil samples were suspended in 150 mL Erlenmeyer flasks containing 90 mL of sterile distilled water and were incubated at 200 rpm and 27°C for 1 h (Innova 44, New Brunswick Scientific). Then, the soil sample suspensions were diluted in sterile distilled water (up to 10^−4^). After that, 0.5 mL of the diluted suspensions were inoculated into Petri dishes containing ISP2 agar (10 g/L malt extract, 4 g/L yeast extract, 4 g/L glucose and, 20 g/L bacteriological agar) and incubated at 40°C for 72 h. Isolated colonies were transferred to new Petri dishes with fresh ISP2 agar and incubated again at 40°C for 72 h. Axenic cultures were stored at 4°C for further analysis.

### 2.4. Submerged culture to obtain secretomes

Submerged cultures of isolates were established in 250 mL Erlenmeyer flasks containing 50 mL of ISP2 broth. The culture media were inoculated with a single colony of the axenic isolates and incubated at 37°C at 180 rpm for 72 h (Innova 44, New Brunswick Scientific). Then, the culture media were centrifuged at 10,034 x g and 4°C for 20 min (Allegra 64R Centrifuge, Beckman Coulter), and supernatants (cell-free) containing secretomes were stored at −20°C for further chemical analysis or bioassays.

### 2.5. Chemical characterization of secretomes by thin-layer chromatography

The selected secretomes with the highest nematicidal activities were freeze-dried (Yamato DC401 freeze dryer), and metabolites contained in the lyophilized powder were further extracted with methanol. 300 μL of solvent were added to 8 mg of lyophilized secretome and sonicated for 15 min in an ultrasonic bath (Branson 2510). Then, the samples were centrifuged (Eppendorf Centrifuge 5417R) at 9,279 × g and 4°C for 10 min, and the supernatants were collected for TLC analysis. Eight microliters of the extracts were spotted on silica gel plates (10 × 10 cm, TLC Silica gel 60 F_254_, Merck) and eluted with a mobile phase containing chloroform: methanol: distilled water (65:25:4 v/v). After elution, compounds were visualized with UV light (A: 254, and C: 365 nm), ninhydrin (sprayed with 0.1% w/v ethanol and heated at 60°C for 15 min), or iodine vapors.

### 2.6. Molecular identification of bacterial isolates

#### 2.6.1. Genomic DNA extraction

Genomic DNA was extracted from a liquid culture of isolate A81 (further TB197 strain) grown aerobically in LB broth for 24 h at 30°C using the PowerSoil^®^ DNA isolation kit (MO BIO Laboratories Inc.) according to the standard protocol provided by the manufacturer.

#### 2.6.2. PCR amplification of the 16S *rRNA*, *gyrA*, and *groEL* genes

PCR amplification of marker genes was performed using OnePCR^™^ Ultra Supermix with Fluorescent Dye (Bio-Helix) according to the methodology described by Ben Gharsa et al. (2021) with some modifications. The primer pairs used for the 16S *rRNA*, *gyrA*, and *groEL* genes are detailed in Table 1. For all markers, a Studio^™^ 5 Real-Time PCR System was used (Applied Biosystem by Thermo Fisher Scientific), with a unified PCR program that consisted of (1) an initial denaturation phase at 95°C for 5 min; (2) 30 cycles of denaturation at 95°C for 40 s, annealing at 56°C (for the 16S region) or 55°C (for the other genes) for 1 min and elongation at 72°C for 30 s; and (3) final elongation at 72°C for 2 min.

**Table 1:** Primers used for the molecular identification of bacterial strains.

Gene	Primer	Primer Sequence (5′-3′)	Reference
*16S rRNA*	27F	AGAGTTTGATCMTGGCTCAG	Ben Gharsa et al. (2021)
	1492R	TACGGYTACCTTGTTACGACTT	
*gyrA*	42F	CAGTCAGGAAATGCGTACGTCCTT	
	1066R	CAAGGTAATGCTCCAGGCATTGCT	
*groEL*	550F	GAGCTTGAAGTKGTTGAAGG	
	1497R	TGAGCGTGTWACTTTTGTWG	

The amplified 16S *rRNA*, *gyrA* and *groEL* products were confirmed by horizontal agarose gel electrophoresis with 1Kb Plus DNA ladder RTU (Bio-Helix). The gels were visualized on a UVP^®^ High-Performance UV Transilluminator (Thermo Fisher Scientific).

#### 2.6.3. Sequencing and phylogenetic analysis of the 16S *rRNA*, *gyrA*, and *groEL* genes

The PCR products were purified and analyzed by PSOMAGEN. Raw sequence data were combined into a single consensus sequence for each marker gene using the MEGA program version 10.0.05 (Kumar et al., 2018). The consensus sequences obtained were used as queries in GenBank database searches using the BlastN algorithm (NCBI GenBank database). Phylogenetic analysis was performed using MEGA software version 10.0.05 (Kumar et al., 2018). Evolutionary distances were calculated by Kimura’s two-parameter model (Kimura, 1980). Phylogenetic trees based on the 16S *rRNA*, *gyrA*, and *groEL* sequences of the isolates and different isolates retrieved from NCBI GenBank^1^ were constructed using the neighbor-joining method with bootstrap values based on 1,000 replicates (Saitou and Nei, 1987). The obtained 16S *rRNA*, *gyrA*, and *groEL* gene sequences of the isolates were submitted to the GenBank database.

### 2.7. Collection and synchronization of larval and egg stages of phytopathogenic nematodes

Nematode synchronization was performed based on the Zhao et al. (2020) methodology with some modifications. Tomato roots highly infested with *Meloidogyne incognita*, *M. enterolobii* or *Radopholus similis* were collected and vigorously washed with water to remove any adhered soil. The roots were carefully cut into 2–4 cm long pieces and placed into a blender containing a NaClO solution (2% w/v) to grind for 30 s. The crushed roots were rinsed with tap water and passed through 100-, 325-, and 500-mesh sieves. Second-stage (J2) juveniles of PPNs retained on the 325-mesh sieves and eggs retained in the 500-mesh sieves were suspended in sterile tap water and stored at 15°C for further analysis. On average, approximately 200 J2s or eggs per milliliter were collected by this procedure (five counts visualized at 10X, MOTIC, AE 2000, inverted microscope).

### 2.8. *In vitro* screening for nematicidal activity

*In vitro* nematicidal activity bioassays employing *M. incognita* J2 larvae in aqueous suspension were performed according to the methodology of Ala et al. (2020) with some modifications. Twenty microliters of secretomes obtained from submerged cultures were added to 96-well flat-bottom sterile polystyrene microplates (Corning^®^ Costar^®^ 3,595) containing 40–60 J2 larvae suspended in 200 μL of sterile tap water per well. Subsequently, the plates were sealed with parafilm and incubated at 25°C in the dark for 48 h, and motile and immotile larvae (considered death) were counted at 10X amplification (MOTIC, AE 2000, inverted microscope).

All experiments were performed considering three biological replicates (with three technical repeats each). Nematicidal activity was estimated according to Equation (1).


(1) $$\%\mathrm{Mortality}=\frac{\mathrm{dead}\;J2}{\;\mathrm{total}\;J2}*100\%$$

### 2.9. Nematicidal activity evaluation of secretomes in field assays

The evaluation was performed on a tomato (*Solanum lycopersicum* L.) crop within the presence of a shade mesh (∼50% reduction of sunlight radiation) at Agroindustrias Tombell (Culiacan, Sinaloa-Mexico). The experimental area was selected based on previous analyses of the nematode populations, and areas with the highest level of *M. enterolobii* infestation were selected. Tomato seedlings of the commercial Dionysus^®^ hybrid (Ahern Seeds) were transplanted into cultivation plots, and the following three treatments were applied: (1) conventional management (undisclosed by the farmer), (2) secretome of the selected isolate from *in vitro* screening, and (3) Verango^®^ (fluopyram, 41.7%), as a positive control. The secretome (cell free supernatant) of the selected isolate was applied at a concentration of 8 L/ha, while Verango^®^ was applied at 1 L/ha. A total of 12 applications were made using a drench system with intervals of 8 days between them for a period of 90 days. Each experimental unit consisted of three plots (1.80 m between them) and was 50 m long (270 m^2^ per treatment). Only the central plot of each treatment was evaluated to avoid the influence of adjacent treatments.

To evaluate the root damage produced by nematodes, root washings were performed at 30, 60, and 90 days after transplantation, for which 10 plants were selected randomly from the central plot of each treatment. The galling index (GI) in tomato roots was determined based on the visual scale proposed by Baker (1978) (Supplementary Table S1; Supplementary Figure S1) in a range of 0–5, where 0 represents 0% galling and 5 represents greater than 80%.

### 2.10. Endospores production

Five hundred milliliter Erlenmeyer flasks, containing 200 mL of sterile Soy based medium (15 g/L soybean flour, 5 g/L dextrose, 5 g/L cornstarch, 1 g/L K_2_HPO_4_, 1 g/L of KH_2_PO4, 0.3 g/L MgSO_4_*7H_2_O, 0.02 g/L FeSO_4_*7H_2_O, 0.02 g/L ZnSO_4_*7H_2_O and 1 g/L CaCO_3_; pH: 7) were aseptically inoculated with a single bacterial colony obtained from a Petri dish. The flasks were incubated at 37°C, 200 rpm, during 72 h. Finishing the incubation period, a heat treatment was employed by subjecting the culture to 80°C for 15 min to eliminate selectively vegetative cells, following the procedure described by Vignesh et al. (2022).

Subsequently, the endospore suspension was added with 10% (w/v) of maltodextrin as a carrier (Bhagwat et al., 2020; Vignesh et al., 2022) and dried using a laboratory spray dryer (Yamato ADL3115) at 110°C and 75°C as inlet and outlet air temperature, respectively, obtaining a fine powder containing around 1 × 10^8^ endospores/g. Endospores were quantified, resuspending the powder in water and employing two methods: (a) using a Newbauer chamber (40X); (b) by plate count, according to Bolanos-Carriel and Vazquez Soto (2022) method. Endospores morphological and wall characteristics were verified through the application of the Endospore Stain Protocol, recommended by the American Society for Microbiology (Hussey and Zayaitz, 2016). The endospores powder was employed further in greenhouse and open field bioassays.

### 2.11. Biological control in greenhouse assays

The assays were performed at Centro de Investigación en Alimentación y Desarrollo (Culiacán, Sinaloa, Mexico) in the spring of 2020 (March and April). Taureg hybrid tomato seeds were sown directly in plastic pots under aseptic conditions at a greenhouse temperature of ~25°C, relative humidity of ~60% and 16 h of daylight. A cluster of 4 to 6 seeds was sown approximately 1.5 inches deep in a 15-cm-wide pot, which was filled with 1.0 kg of a high-quality well-drained potting mix. The plants were watered every 3–4 days, and when they were 30 days old, a suspension of *M. enterolobii* eggs (approximately 1,500 eggs per pot) was inoculated using a sterile micropipette following standard inoculation procedures (Naz and Khan, 2013).

To evaluate the biological control in greenhouse assays, 400 mL of (a) water (negative control), (b) selected strain endospores suspension (1 × 10^6^ endospores/mL; prepared resuspending 1 g of dried spores (1 × 10^8^) in 100 mL of water), and (c) Verango^®^ (fluopyram, 41.7%, as a positive control, 1 L/ha) were applied by irrigation to 20 plants postinoculation of the *M. enterolobii* eggs. After 30 and 60 days of nematode inoculation, five plants from each treatment were picked randomly, and the roots were carefully washed to remove soil remnants. The galling index was measured to evaluate the biological efficacy of the treatments applied according to the aforementioned visual scale proposed by Baker (1978) (Supplementary Table S1; Supplementary Figure S1). The number of eggs per pot was also recorded by counting under an inverted microscope to determine the reproduction factor (RF) of *M. enterolobii*, according to Equation (2).


(2) $$\mathrm{RF}=\frac{\mathrm{Final number of eggs}}{\mathrm{Initial number of eggs}\;}$$

### 2.12. Biological control in open field assays

#### 2.12.1. Root-knot nematodes

Biological control of the root-knot nematode *M. incognita* was conducted in Calvillo, Aguascalientes, Mexico, using tomato plants (*Solanum licopersicum*) of the Optimax variety during May and June 2022. A randomized complete block design was employed, where 100 mL (per plant) of (a) water as a negative control, (b) selected isolate endospores (1 × 10^8^ endospores/g; 4 kg/ha) and (3) Lila Plus^®^ as a positive control (*Purpureocillium lilacinum*, 0.48 kg/ha) were applied by uniform drenching after 4 weeks of tomato crop growth in four replicate plots (1.8 × 4 m) per treatment. At 15 and 30 days after the treatments, five plants were extracted from each plot (20 plants per treatment), and the galling index was determined by the visual scale described previously (Supplementary Table S1; Supplementary Figure S1). Additionally, to determine the population density of *M. incognita* J2 at the beginning, middle, and end of the assay, 100 g of roots of 4 different plants for each treatment were obtained by 4 zigzag samplings and processed for the extraction and quantification of *M. incognita* (J2, five counts visualized at 10X, MOTIC, AE 2000, inverted microscope) as described previously.

#### 2.12.2. Burrowing nematodes

The biological control of the burrowing nematode *Radopholus similis* was conducted in Cihuatlan, Manzanillo-Mexico using banana plants (*Musa* sp.) of the gran nain cultivar during May and June 2022. A randomized complete block design was employed, where 100 mL of (a) water (negative control), (b) *B. paralicheniformis* TB197 isolate (1 × 10^8^ endospores/g; 4 kg/ha), and (c) Nemacem^®^ (6 L/ha, positive control) were applied by uniform drenching in four replicate plots (1.8 × 4 m) per treatment. At 15 and 30 days of the assay, five plants were extracted from each plot (20 plants per treatment), and the necrosis index was determined based on a visual scale of 0 to 5 proposed by Coyne et al. (2007) (Supplementary Table S2). To determine the population density of *R. similis* J2 at the beginning, middle and end of the assay, 100 g of roots of 4 different plants for each treatment were obtained by 4 zigzag samplings and processed for the extraction and quantification of *R. similis* J2 as described previously.

### 2.13. Statistical analysis

The results of all experiments are reported as the mean ± standard deviation. One-way analysis of variance (ANOVA) and Tukey–Kramer (95% confidence limit) tests were performed to establish significant differences among treatments using NCSS software (Number Cruncher Statistical System or Windows, Kaysville, UT, United States, version 7.0).

## 3. Results

### 3.1. *In vitro* screening for nematicidal activity from secretomes

**Figure 1:**
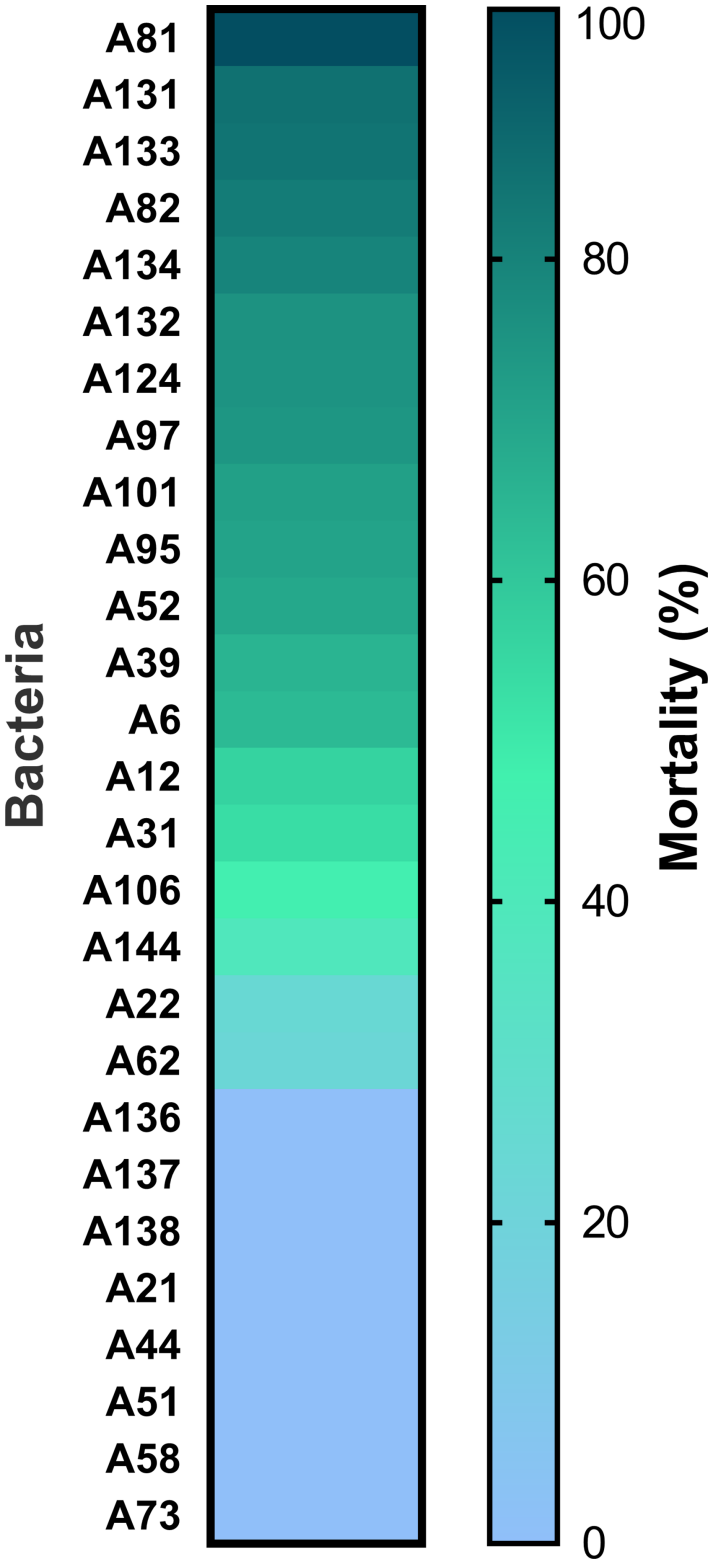
Toxicity of secretomes from bacteria isolated from rhizospheric soil samples from the Sonoran Desert against *M. incognita*. Color bars represent the means of three replicates.

A total of 100 bacterial isolates were isolated from rhizospheric soil samples collected at the Sonoran Desert. Among them, 43 from “La Primavera,” 30 from “Apache,” and 27 from “El Pinacate.” From all isolates, only 27 secretomes showed some nematicidal activity (3–96% mortality *M. incognita* J2 larvae) (Figure 1). Secretomes from the A106, A136, A137, A138, A144, A21, A22, A44, A51, A58, A62, and A73 isolates showed low nematicidal activities (≤50%), those from the A12, A31, A39, A52, and A6 isolates presented moderate activities (50–70%), those from the A101, A124, A131, A132, A133, A134, A82, A95, A74, and A81 isolates were highly effective (70–96%). Among these, A81 isolate showed the highest and consistent nematicidal activity (96%), it was chosen as the most promising microorganism for PPNs control; thus, a comprehensive characterization of this isolate, including its molecular identification, *in vivo* effectiveness assays (both endospores and secretome), and chemical characterization of its metabolites, were performed.

### 3.2. Molecular identification of the selected isolate

**Figure 2:**
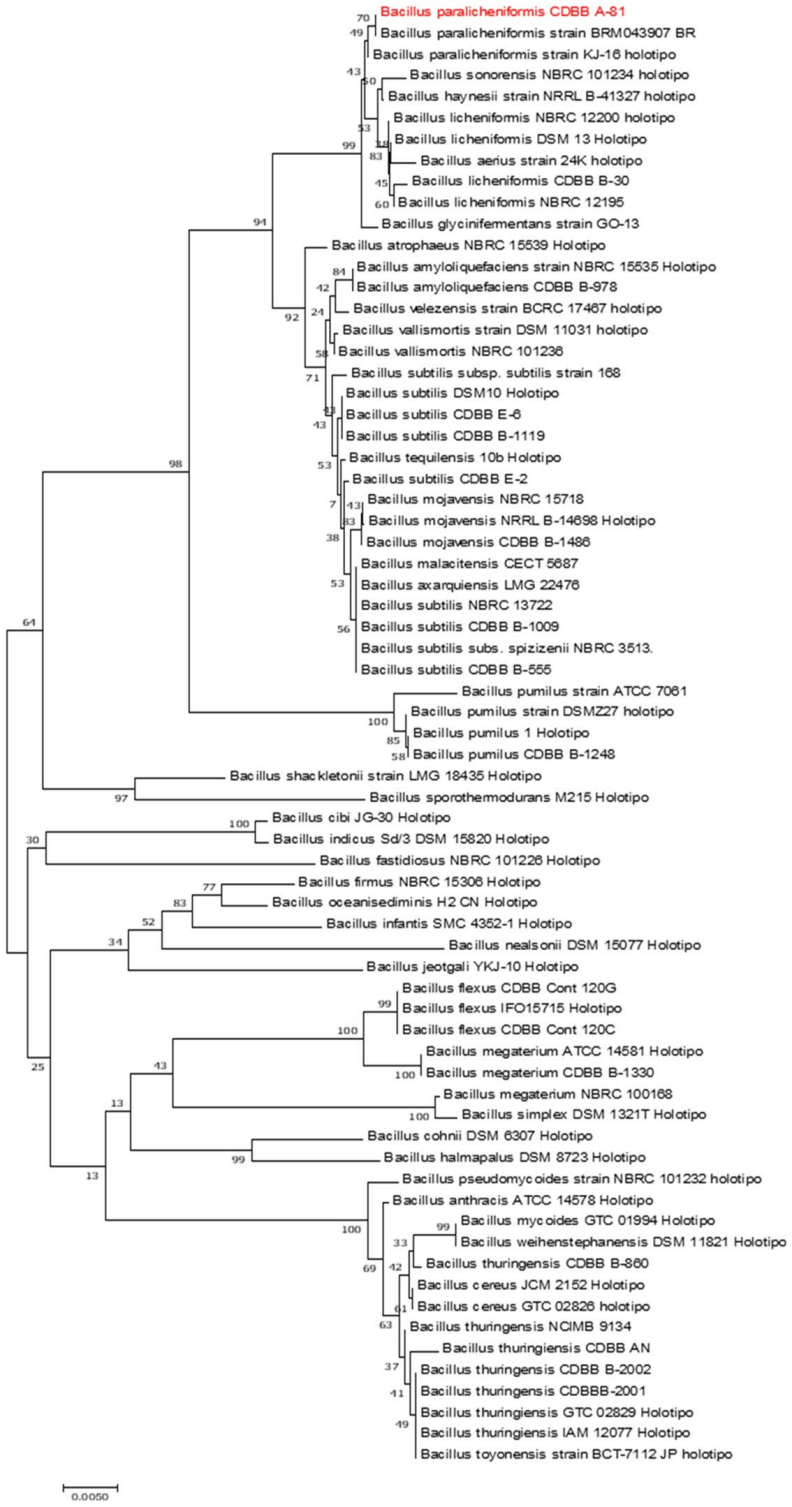
Phylogenetic tree of the representative TB197 isolate and *Bacillus* species from NCBI GenBank based on the 16S *rRNA* gene. The sequence was constructed using the neighbor-joining method with 1,000 replicates. The bar indicates 0.005 substitutions per nucleotide position.

According to the 16S *rRNA* (Figure 2) and the housekeeping *gyrA* and *groEL* gene sequences (Supplementary Figures S2, S3), the A81 isolate showed 99.27–100% identity to *Bacillus paralicheniformis*. The percent identity values and the accession numbers of the sequences are listed in Table 2. The A81 isolate was deposited at the National Genetic Resources of Mexico, CNRG, and renamed as *Bacillus paralicheniformis* TB197 strain.

**Table 2:** Molecular identification and genotyping of *Bacillus paralicheniformis* TB197 based on 16S *rRNA*, *gyrA*, and *groEl* gene sequences.

Gene	Number of nucleotides	Identity (%)	Accession number
*16S rRNA*	1,466	100.00	OP962217
*gyrA*	954	99.27	OQ127874
*groEL*	938	99.68	OQ127873

### 3.3. Chemical characterization of secretomes by thin-layer chromatography

**Figure 3:**
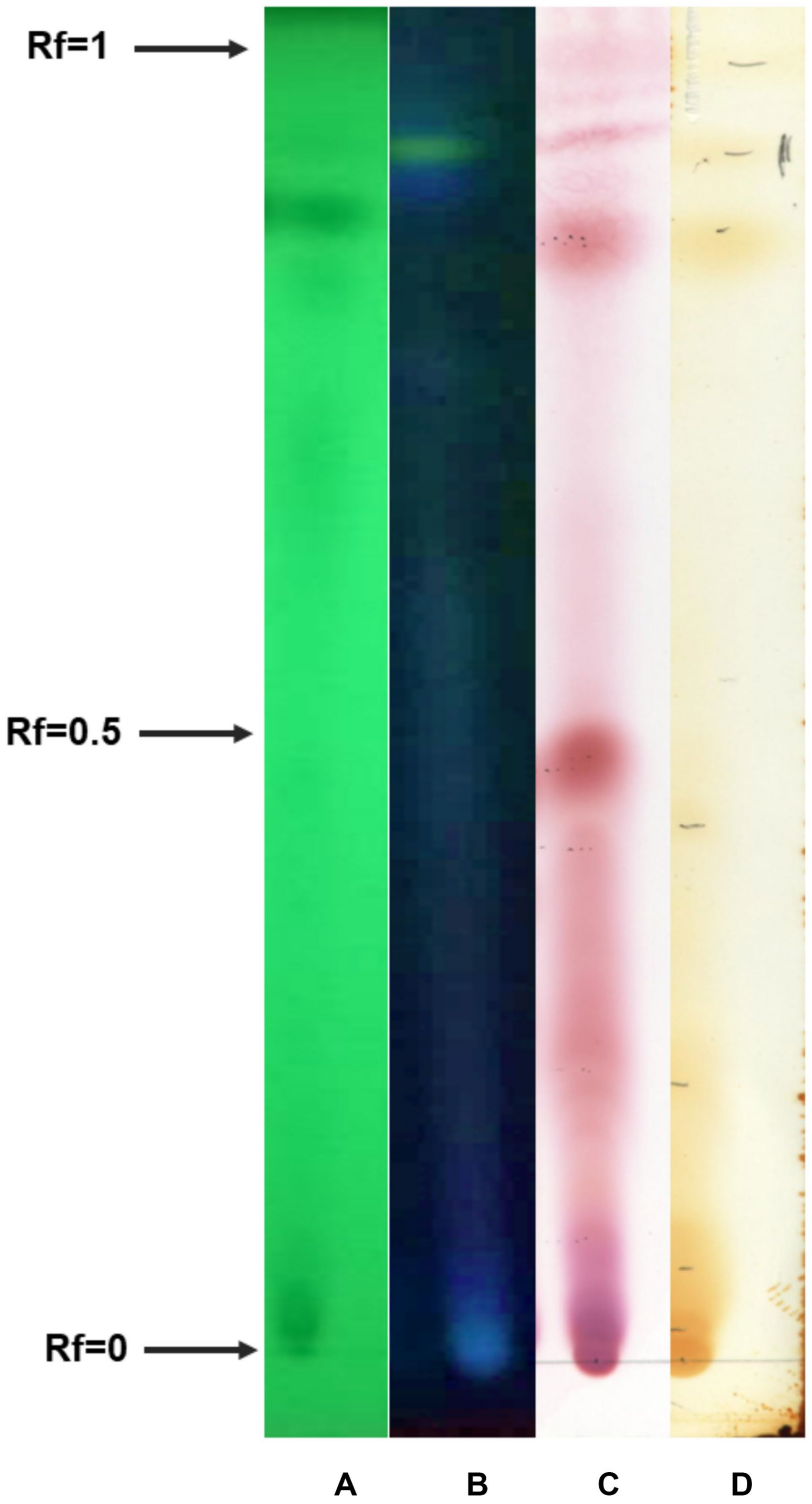
Thin-layer chromatogram of the *B. paralicheniformis* TB197 secretome visualized with **(A)** UVA light, 254 nm; **(B)** UVC light, 365 nm; **(C)** ninhydrin (0.1%); and **(D)** iodine vapors. Rf, retard factor.

The secretome of the *B. paralicheniformis* TB197 strain was analyzed by thin-layer chromatography (TLC) employing UV light (to reveal conjugated pi bonds, such as aromatic compounds), ninhydrin (to reveal peptides and free amino groups) and iodine vapors (to reveal unsaturated compounds, particularly fatty acids), as shown in Figure 3. TLC plates demonstrated the presence of high-polarity compounds with low distance traveled (Rf from 0 to 0.4), active to UV light (Figures 3A,B), and high reactivity to ninhydrin and iodine vapors (Figures 3C,D, respectively). On the other hand, at least one compound of medium polarity (Rf near 0.5) was revealed with ninhydrin and iodine vapors (Figures 3C,D) but was inactive or slightly active to UV light (Figures 3A,B). Finally, two compounds of low polarity with an Rf of 0.8 and 0.9 were visualized employing UVC and UVA, respectively (Figures 3A,B), and both were reactive to ninhydrin and iodine vapors (Figures 3C,D, respectively).

### 3.4. Secretome nematicidal activity in field assays

The effect of the secretome of the TB197 strain on the galling index (GI) and the percentage of infestation severity on tomato roots caused by *M. enterolobii* was evaluated in field experiments and compared with conventional management (employed by the owner of the field and kept confidential) and the application of a chemical nematicide as a positive control (fluopyram, 41.7% w/v). The three treatments suppressed the rate of galling (0) caused by *M. enterolobii* during the 60 days of application. At 90 days of the experiment, no differences were observed (*p* ≥ 0.05) in the treatments, where the secretome of the TB197 strain (GI: 0.2; Severity (%): 4) and the conventional management (GI: 0.2; severity (%): 4) were applied. In both cases, the progress of the infection was slight, with a GI and severity percentage less than 0.5 and 5%, respectively, while the positive control (fluopyram) completely suppressed the galling and damage caused by *M. enterolobii.*

### 3.5. Biological control in greenhouse assays

**Figure 4:**
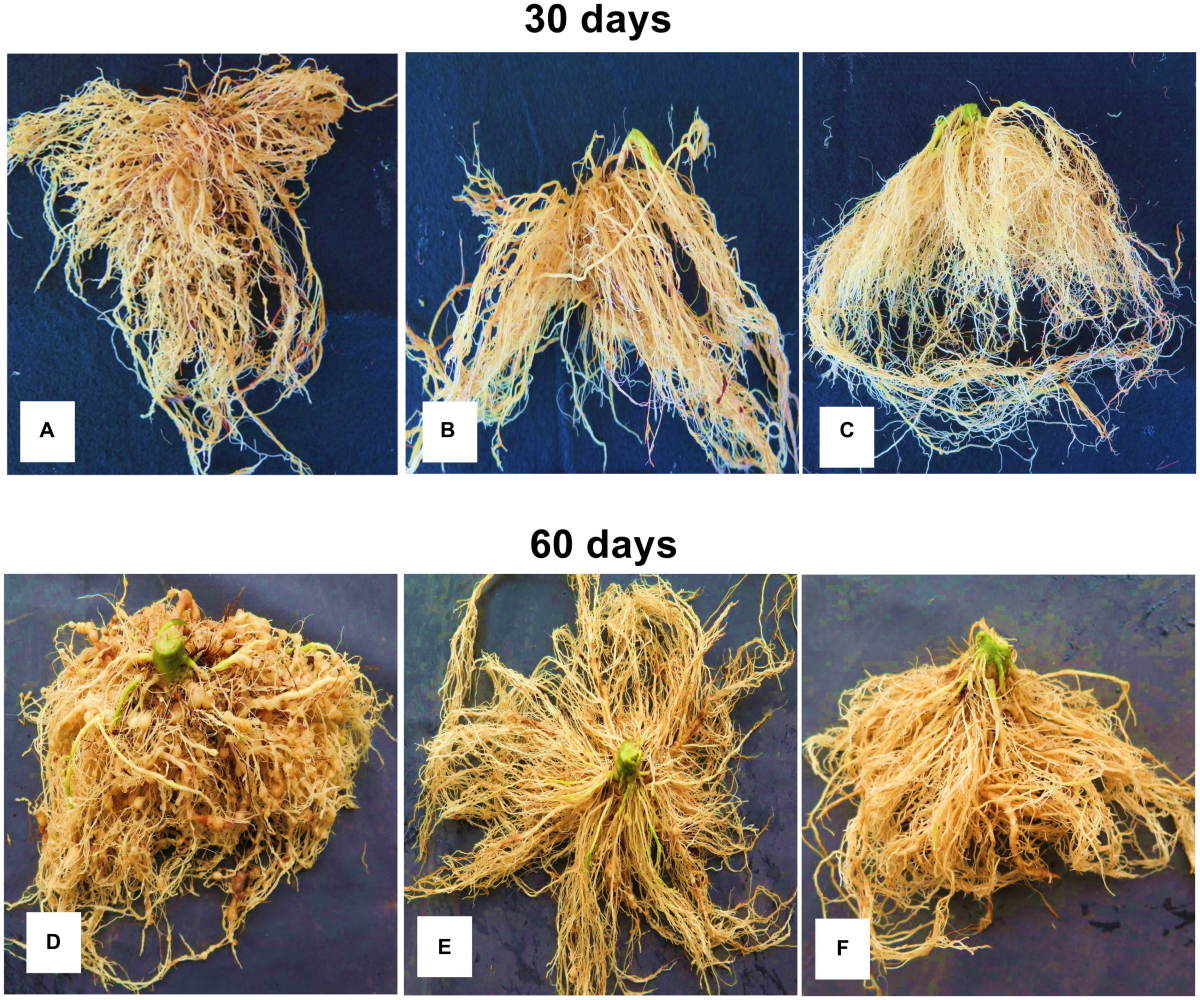
Gall formation on tomato roots caused by *M. enterolobii* in a greenhouse assay after 60 days of treatment. **(A,D)** Negative control (water); **(B,E)** *B. paralicheniformis* TB197 endospores; **(C,F)** positive control (fluopyram).

A higher and progressive nematode infestation was observed in the control (water) at the first (30 days) and second evaluation (60 days) (Figures 4A,D), resulting in a galling index (Table 3) up to 10-fold higher than the application of *B. paralicheniformis* TB197 endospores and the chemical nematicide (*p* ≥ 0.05). *Bacillus paralicheniformis* TB197 endospores effectively suppressed the infestation of *M. enterolobii* in tomato roots (Figures 4B,E) in a similar manner (*p* ≤ 0.05) as the chemical nematicide (Figures 4C,F) at 30 and 60 days of application. The reproduction factor of *M. enterolobii* in tomato roots (Table 3) treated with the chemical nematicide and TB197 endospores were similar between them (*p* ≤ 0.05) but 116-fold lower than that of the negative control (*p* ≥ 0.05).

**Table 3:** Effect of treatments on *M. enterolobii* (greenhouse test) infestation of tomato plants measured through the galling index (GI), infestation severity, population density and reproduction factor.

Treatments	30 days	60 days			
	GI	Severity (%)	GI	Severity (%)	Reproduction factor
Water	1.80 ± 0.83^b^	8.2^b^	4.2 ± 0.75^b^	64.4^b^	7.00 ± 0.006^b^
*B. paralicheniformis* TB197 endospores	0.20 ± 0.44^a^	0.2^a^	0.8 ± 0.75^a^	0.8^a^	0.06 ± 0.002^a^
Fluopyram	0.00 ± 0.00^a^	0.0^a^	0.4 ± 0.49^a^	0.4^a^	0.06 ± 0.001^a^

Experimental data are represented as the mean ± standard deviation for five individual experiments. The different superscript lowercase letters on columns indicate significant differences for each assay according to the Tukey–Kramer test (*p* < 0.05).

### 3.6. Biological control in open-field assays

**Figure 5:**
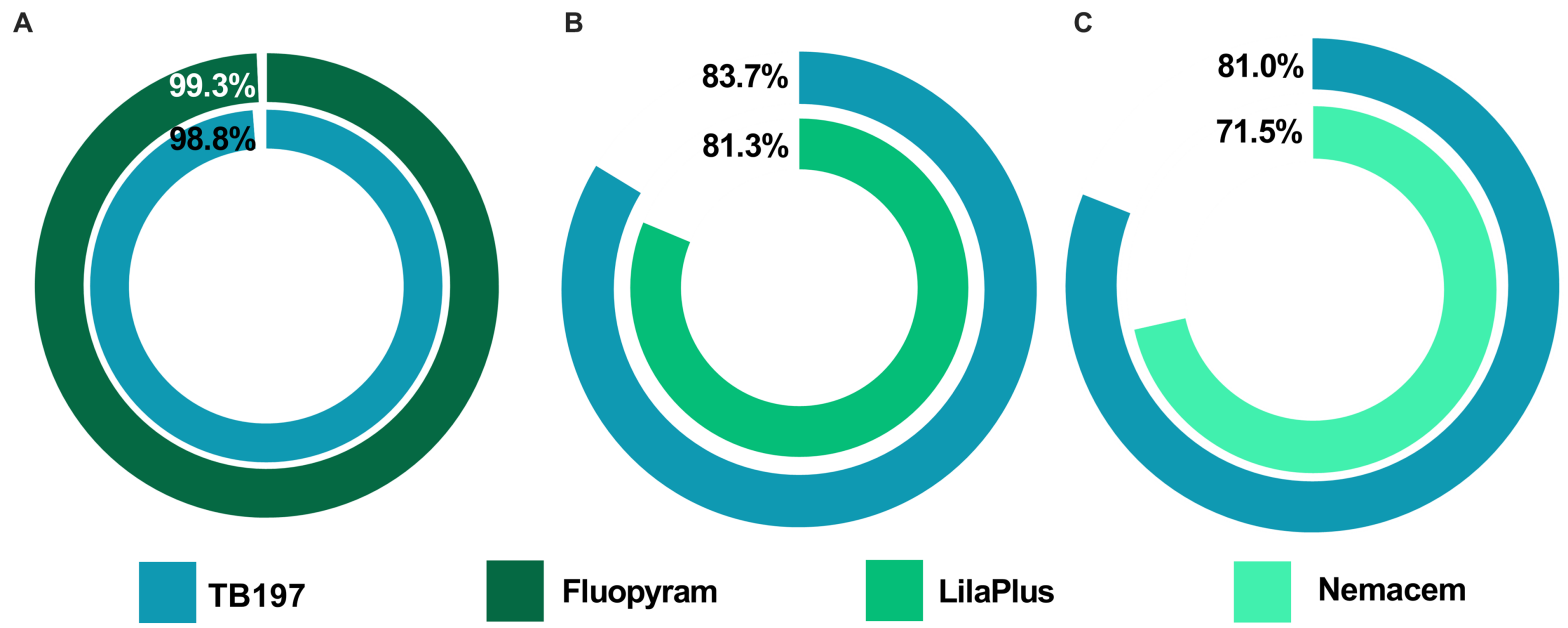
Control efficacy on phytopathogenic nematodes using endospores of the *B. paralicheniformis* TB197 strain and commercial nematicides. **(A)** Reduction in the galling severity (%) produced by *M. enterolobii* (greenhouse test); **(B)** reduction in the galling severity (%) produced by *M. incognita* (field test) and **(C)** reduction in the necrosis percentage produced by *R. similis* (field test). The reduction percentages are based on the negative control (water).

Like greenhouse assay, a higher and rapid infestation of the root-knot nematode (*M. incognita*) was observed in the control (water) during the open-field assay (15 and 30 days), while *Bacillus paralicheniformis* TB197 endospores and Lila-Plus^®^ (*Paecilomyces lilacinus* 3% w/w) effectively reduced the infection parameters evaluated in tomato plants (*p* ≤ 0.05) without significant differences between them (Table 4). With both treatments, the GI, severity percentage and, population density of *M. incognita* were up to 2-, 5-, and 7-fold lower than those of the control, respectively (Table 4). Additionally, based on the reduction in galling severity, the efficacy of TB197 and the positive control in controlling *M. incognita* was 83.7 and 81.3%, respectively (Figure 5B).

**Table 4:** Effect of treatments on *M. incognita* infestation of tomato plants (field test) measured through the galling index (GI), infestation severity and population density.

Treatments	15 days			30 days		
	GI	Severity (%)	Population density	GI	Severity (%)	Population density
Water	3.2 ± 0.35^b^	21^b^	80.8^b^	3.5 ± 0.28^b^	43^b^	172.3^b^
*B. paralicheniformis* TB197 endospores	1.4 ± 0.44^a^	5^a^	11.5^a^	1.6 ± 0.32^a^	7^a^	23.8^a^
Lila Plus^®^ (*P. lilacinus*)	1.3 ± 0.52^a^	4^a^	14.5^a^	1.7 ± 0.47^a^	8^a^	28.0^a^

Experimental data are represented as the mean ± standard deviation of five individual experiments. The different superscript lowercase letters on columns indicate significant differences for each assay according to the Tukey–Kramer test (*p* < 0.05).

The *B. paralicheniformis* TB197 endospores also showed consistent nematicidal activity against the burrowing nematode *R. similis* in banana crops. TB197 strain and Nemacem^®^ (aqueous extract from *Tagetes erecta*, alpha terthienyl 10% w/w) effectively reduced the infection caused by *R. similis* (Table 5) without significant differences between them (*p* ≥ 0.05) during the open-field assay (15 and 30 days). Both reduced necrosis of roots and nematode population density up to 4.4- and 2.6-fold, respectively, compared with the control (water), with which banana crops showed a faster *R. similis* infestation. Additionally, based on the reduction in the necrosis percentage, the efficacy of TB197 and the positive control in controlling *R. similis* was 81 and 71.5%, respectively (Figure 5C).

**Table 5:** Effect of treatments on *Radopholus similis* infestation of banana plants (field test) measured through the necrosis percentage and population density.

Treatments	15 days		30 days	
	Necrosis (%)	Population density	Necrosis (%)	Population density
Water	16.9 ± 1.19^b^	53.3^b^	26.3^b^	99.0^b^
*B. paralicheniformis* TB197 endospores	3.8 ± 1.23^a^	19.8^a^	5.0^a^	28.8^a^
Nemacem^®^ (Alpha terthiernyl)	4.4 ± 0.98^a^	23.5^a^	7.5^a^	30.3^a^

Experimental data are represented as the mean ± standard deviation of five individual experiments. The different superscript lowercase letters on columns indicate significant differences for each assay according to the Tukey–Kramer test (*p* < 0.05).

## 4. Discussion

Harsh abiotic conditions existing in desert environments (e.g., extreme temperatures and radiation, water scarcity, erosion, low organic matter content, and sedimentation, among others) negatively impact living organism development especially in plants and microorganisms (Alsharif et al., 2020). However, several species have developed adaptations to remain metabolically active (Schultz and Soares, 2020) and to be able to proliferate under these adverse conditions. Such is the case for the Sonoran Desert, which possesses the greatest biodiversity of flora and fauna among deserts worldwide, although the temperatures in summer can exceed 45–50°C (Zolotokrylin et al., 2020). Desert soils worldwide possess a huge diversity of microorganisms dominated by Actinobacteria (36.8%), Proteobacteria (23.8%), Firmicutes (8.6%), and Acidobacteria (5.5%) (Dean et al., 2020). According to this microbial diversity distribution and the well-known potential of Actinobacteria as control agents (Bhatti et al., 2017; Cruz Silva et al., 2022); ISP2 medium was employed in the first screening, looking for isolates belonging to this phylum or to Firmicutes (Tian et al., 2022). Since, although ISP2 is considered as a selective medium for actinomycetes, it allows the growth of some other bacterial genera, such as *Bacillu*s, too (Paz et al., 2016; Zhou et al., 2019).

Specifically, regarding the Sonoran Desert, there is scarce information about rhizospheric and soil microbiota composition and the biotechnological potential from their microorganisms. Sutto-Ortiz et al. (2017) and Bacame-Valenzuela et al. (2015) reported the isolation of thermotolerant actinomycetes and fungal strains capable of synthesizing enzymes of industrial interest, such as phospholipase A and feruloyl esterases, respectively. Additionally, Camacho-Ruiz et al. (2020) reported the isolation from Sonoran Desert soils of *Streptomyces*, *Bacillus*, *Aspergillus*, and *Aureobasidium* strains able to produce gastrointestinal lipase inhibitors. Moreover, Baqueiro et al. (2019) reported the biotechnological potential of *Bacillus* strains capable of producing stable proteases. Regardless of agricultural applications, some plant growth-promoting rhizobacteria (PGPRs), such as *Azospirilum brasilense* and *Bacillus pumilus*, have been isolated (Bashan et al., 2012; Galaviz et al., 2018), but there are no reports focusing on PPNs control. Indeed, this is the first work to explore and demonstrate the potential of microorganisms from the Sonoran Desert for the control of PPNs.

In the current work, from 100 thermotolerant bacteria isolated from rhizospheric soils collected in the Sonoran Desert, the secretomes from 27 of them showed some nematicidal activity *in vitro* (3–96%) against *M. incognita*. Of these isolates, 10 produced high mortality in *M. incognita* larvae (70–90%), but the secretome from the TB197 strain produced the highest (>95%) (Figure 1). Consequently, it was selected for identification and evaluation in greenhouse and open-field tests. Sequence analysis of the 16S *rRNA* gene had the most significant impact on the identification and taxonomic classification of the TB197 strain. Even so, analyses of the housekeeping gene sequences *gyrA* and *groEL* were also included because previous studies mention difficulties in the differentiation and phylogenetic relationships of certain groups of *Bacillus* based on 16S *rRNA* gene sequences due to their similarities (Table 2). Additionally, the sequencing of housekeeping genes has been shown to be more appropriate than 16S *rRNA* sequencing for discriminating certain species of *Bacillus* (Ozdemir and Arslan, 2022). Therefore, the use of these housekeeping genes provides a high resolution between species closely related to groups of *Bacillus*, as they are generally more conserved between bacterial species than the 16S *rRNA* gene (Liu et al., 2013; Franco-sierra et al., 2020). This information allowed the robust identification of the TB197 strain as *Bacillus paralicheniformis*.

In agriculture, many strains from the *Bacillus* genus have been employed in biofungicides and biofertilizer formulations due to their biological activities, including antagonism against phytopathogenic agents, such as bacteria, fungi and nematodes; induction of plant systemic resistance against phytopathogens; and promotion of plant growth and development (Villarreal-delgado et al., 2018; Valenzuela-Ruiz et al., 2019). Furthermore, *Bacillus* is one of the three most important genera for PPNs control by several mechanisms: direct parasitism, antibiosis, reduction in plant root penetration and competition for essential nutrients (Engelbrecht et al., 2018). Antibiosis has been identified as the main control mechanism of PPNs through the production of antibiotics and other secondary metabolites (lipopeptides, siderophores, and endotoxins, among others) and lytic enzymes secreted during growth and the stationary phase (Xiong et al., 2015; Gao et al., 2016; Engelbrecht et al., 2018; Liu et al., 2020a). Nevertheless, information on secondary metabolites produced by *B. paralicheniformis* with nematicidal activity is almost nonexistent. Valenzuela-Ruiz et al. (2019) reported that the draft genome of *B. paralicheniformis* TRQ65, isolated from wheat fields in the Yaqui Valley, Mexico, revealed the presence of putative genes related to osmotic and oxidative stress responses and auxin, lipopeptides, siderophores and antibiotic biosynthesis.

For preliminary chemical characterization, extracts obtained by lixiviation of freeze-dried supernatants with methanol were employed. TLC plates from samples obtained by methanolic extraction are shown in Figure 3, revealing the presence of several fractions with different Rf values. The spots from TLC plates containing the methanolic extract can be visualized by different methods (UV and stains). Almost all of them were reactive to ninhydrin and iodine vapors (Figures 3C,D), but only some of them were active to UVA and/or UVC (Figures 3A,B) with slight activity for UV light but high activity for iodine vapors and ninhydrin (Figure 3). The reactivity to ninhydrin suggests the presence of peptide-natural compounds (Dlamini et al., 2020) and probably molecules that contain unsaturated fatty acids due to reaction with iodine vapors (Meyers and Meyers, 2008). In the case of some UV-active spots, the molecules could include aromatic compounds, such as aromatic amino acids.

The information provided by TLC analyses fits with the description of siderophores and cyclic lipopeptides usually produced by *Bacillus* species and the presence of putative genes to produce them, according to the draft genome of *B. paralicheniformis* TRQ65 (Horak et al., 2019; Valenzuela-Ruiz et al., 2019). In general, lipopeptides contain a hydrophobic fatty acid chain bonded to a hydrophilic cyclic peptide. According to the nature of the cyclic peptide, lipopeptides produced by *Bacillus* species, are generally classified into three families: surfactin, fengycin, and iturin (Falardeau et al., 2013; Yang et al., 2015). Members from the fengycin and iturin families usually contain aromatic amino acids in their peptide ring, such as tyrosine (Zhao et al., 2017), which would explain the UV activity of some compounds (Figure 3). Molecules with Rf values between 0 and 0.3 or 0.2 and 0.4 could belong to the fengycin and iturin families, respectively, as mentioned by Geissler et al. (2016). On the other hand, low-molecular-weight metal-chelating compounds of the hydroxamate and catecholate types, which are involved in the uptake of iron (siderophores) by microorganisms, have been reported as metabolites produced by several *Bacillus* species (Sansinenea and Ortiz, 2011). The results of the TLC analysis strongly suggest that secondary metabolites synthesized by the *B. paralicheniformis* TB197 strain could be siderophores and/or cyclic lipopeptides. However, an exhaustive chemical characterization of its secretome is currently ongoing to verify this hypothesis.

This study demonstrated the potential of the secondary metabolites contained in the secretome from *B. paralicheniformis* TB197 to effectively control infections caused by *M. enterolobii* in open-field assays. The GI and the infection severity percentage observed in tomato roots were very low and similar to the conventional management performed by the farmer. In this sense, there are few studies evaluating the effectiveness of secretomes of *Bacillus* species for the control of PPNs in agricultural fields. For example, Liu et al. (2020b) showed that the application of 16 mL/m^2^ of various secretomes of *Bacillus* strains in tomato roots resulted in a GI of 1, while the GI reported in the present work by employing the secretome of the TB197 strain was 0.2. Therefore, the secretome of *B. paralicheniformis* TB197 appears to be more effective as a lower concentration (0.8 mL/m^2^) was applied and obtained better GI results.

However, additional work over the application of secretomes regarding biosafety, doses, application times, optimization of culture medium, culture conditions and the exhaustive characterization of their bioactive metabolites is required. Although the chemical nematicide fluopyram (pyridinyl-ethyl-benzamide) was highly effective in suppressing infection by *Meloidogyne* species, there are reports that this chemical shows a reversible inhibition phenomenon that could lead to possible crop infestation even after application (Watson, 2022). In addition, fluopyram is reported to be a recalcitrant compound that modifies the general soil structure and the activity and function of beneficial soil microorganisms (Zhang et al., 2014). Consequently, as an advancement toward sustainable agriculture, the secondary metabolites contained in the secretome of the TB197 strain appear to be a promising alternative to chemical nematicides.

Although bacterial metabolites look promising for PPNs control, the use of cells or endospores from *Bacillus* species is the most common way for formulating commercial products (Zhou et al., 2016; Engelbrecht et al., 2018). The use of live cells and/or spores offers several additional advantages over the use of metabolites, such as the ability to combine multiple mechanisms, like interference in nematode-host recognition, competition for nutrients, promotion of plant growth, and induction of systemic resistance (Chinheya et al., 2017; Engelbrecht et al., 2018; Ramírez-Cariño et al., 2020). Additionally, endospores are preferred to prepare commercial formulations because they are highly resistant to adverse environmental conditions, making them suitable for long-term storage and convenient for formulating powdered products for agricultural application (Zhou et al., 2019). In this study, the capacity of *B. paralicheniformis* TB197 to control PPNs was evaluated for the first time. In the greenhouse assay, both the TB197 strain and fluopyram demonstrated a control efficacy of *M. incognita* greater than 98% based on the reduction in galling severity (Figure 5A).

On the other hand, several studies mention that success of controlling PPNs at the greenhouse level does not guarantee bacterial performance at the field level (Tian et al., 2007; Abdel-Salam et al., 2018; Zhao et al., 2020; Wang et al., 2021), usually because strains are affected by unfavorable factors, such as climatic changes, physical and chemical properties of soil and the competition of native microorganisms (Wang et al., 2021). However, our results show the consistent ability of *B. paralicheniformis* TB197 to control PPNs even at the field level. One assay was performed against root-knot PPN comparing the bacterial effectiveness with a probed commercial biopesticide LilaPlus^®^ (*P. lilacinum*). Both treatments were highly effective and showed consistent results for the control and reduction of the severity of *M. incognita* infection (~84% compared with the control, Figure 5B). Several studies and patents report *Bacillus* cells or endospores as biological control agents, particularly against root-knot nematodes from the *Meloidogyne* genus; however, none of them surpassed the effectiveness reported here for *B. paralicheniformis* TB197. For example, the percent reduction in GI in field assays through the use of *Bacillus altitudinis* AMCC1040 (Wang et al., 2021), *B. subtilis* Bs-1, *B. cereus* Bc-cm103 (Cao et al., 2019), *B. aryabhattai* KMT-4 (Yin et al., 2021), *B. velezensis* Bv-25 (Antil et al., 2021), *B. marisflavi* CRB2 (Tian et al., 2022), *B. subtilis* CRB7 and *B. methylotrophicus* (Gowda et al., 2022) was <70%.

A second assay was performed against the burrowing nematode *R. similis*, a migratory, polyphagous endoparasite with a wide global distribution mainly found in tropical and subtropical regions that infects banana crops. The control effectiveness of the TB197 strain was similar to that of Nemacem^®^, an effective commercial botanical extract from *Tagetes erecta*, employed as a positive control. In both cases, the percentage of root necrosis and the nematode population density were reduced by 81.0 and 71.5% (Figure 5C), respectively, compared to the control (water). The main active metabolite from the botanical extract (Nemacem^®^) is alpha terthienyl, a well-known molecule acting on the central and peripheral nervous systems of nematodes, causing their immobilization and subsequent death (Plantoria, 2022). Although this extract is considered an environmentally friendly alternative, its half-life is very short (~ 4 h, according to the National Institute of Ecology and Climate Change of Mexico^2^), which could be a disadvantage compared with bacterial-based nematicides, that in the case of molecules synthetized by the *Bacillus* genus, are stable at extreme soil pH, temperature and salinity conditions for more than 25 days (Raaijmakers et al., 2010; Engelbrecht et al., 2018). Our results demonstrate that both the bioactive metabolites contained in the secretome of the *B. paralicheniformis* TB197 strain and their endospores can effectively control PPNs infections. They provide an ecological alternative to chemically synthesized nematicides, and can compete successfully with commercial botanical and microbiological bionematicides.

Despite the fact that the TB197 strain was isolated from the desert rhizosphere and showed an optimum growth temperature range between 40 and 45°C (data not shown), its ability to growth in different environmental conditions is highlighted. For example, the greenhouse trials were developed under controlled conditions of temperature and humidity (25°C and 60%, respectively), while the field trials on tomato plants were performed in Aguascalientes, Mexico, where the temperature ranged from 13 to 30°C with a relative humidity of 6.7%. By the contrary, the trials on banana crops were carried out in Colima, Mexico, where the temperature varies from 23 to 31°C with a relative humidity of 81%, higher than greenhouse and field trials conditions. These facts suggest that the TB197 strain has the ability to colonize different crop roots under distinct environmental conditions, possibly due to a unique adaptation mechanism inherent to its niche of isolation. However, further studies are required to establish more precisely the colonization capacity of the TB197 strain under extreme conditions (ongoing studies).

## 5. Conclusion

Plant-associated bacteria from the Sonoran Desert show potential for controlling PPNs. In particular, the new *Bacillus paralicheniformis* TB197 strain exhibited a high capacity for PPNs control *in vitro*, greenhouse and agricultural field tests. Moreover, the TB197 strain successfully proliferated in various rhizospheric soils with different climate, soil conditions and crops, indicating its high adaptability to colonize environments different from its niche of isolation. The effective reduction of root infection by PPNs and the resulting damage, achieved through the application of the secretome or the endospores of TB197 strain, are comparable to those obtained with commercial chemically synthesized and natural nematicides. These results support *B. paralicheniformis* TB197 as a potential control agent for the development of commercial bionematicidal formulations.

## Data availability statement

The datasets generated during and/or analyzed during the current study are available from the corresponding author on reasonable request.

## Author contributions

EC-Q, VC-J, AC-F, FD, and AA-T contributed to the study conception and design. AC-F performed the secretome field and the greenhouse assays. VC-J obtained the secretomes for the *in vitro* nematicidal assays. FD performed the molecular characterization. EC-Q performed the chemical characterization of the secretome, analyzed the data, and wrote the original manuscript. AA-T and VC-J supervised the research and reviewed the manuscript. All authors contributed to the article and approved the submitted version.

## Funding

This work was supported by Grant N° 20624 Innovak Global-CIAD A.C.

## Conflict of interest

The authors declare that the research was conducted in the absence of any commercial or financial relationships that could be construed as a potential conflict of interest.

## Publisher’s note

All claims expressed in this article are solely those of the authors and do not necessarily represent those of their affiliated organizations, or those of the publisher, the editors and the reviewers. Any product that may be evaluated in this article, or claim that may be made by its manufacturer, is not guaranteed or endorsed by the publisher.

## Footnotes

^1^ https://blast.ncbi.nlm.nih.gov/Blast.cgi

^2^ http://www2.inecc.gob.mx/sistemas/plaguicidas/pdf/Alfa%20terthienil.pdf
