# Supplementary material for: Native Bacillus paralicheniformis isolate as a potential agent for phytopathogenic nematodes control
Source: Front Microbiol. 2023 Jul 31;14:1213306. doi: 10.3389/fmicb.2023.1213306 (PMC10425774; doi:10.3389/fmicb.2023.1213306)
Supplement: Supplementary file 1 [file Data_Sheet_1.pdf]

Supplementary Material

A new *Bacillus paralicheniformis* native strain with high potential for phytopathogenic nematodes control

Estefany Chavarria-Quicaño, Victor Contreras-Jácquez, Armando Carrillo-Facio, Francisco De la Torre-Gonzáles, Ali Asaff-Torres**

Corresponding Author: asaff@ciad.mx

1 Supplementary Tables and Figures

Supplementary Table 1. Scale to evaluate the root-knot index caused by Root-knot nematodes

Scale	% Galling
0	0
1	1-10
2	10.1-20
3	20.1-60
4	60.1-80
5	80.1-100

Supplementary Table 2. Scale to evaluate the rate of necrosis caused by Root-injuring nematodes

Scale	% Necrosis
0	0
1	1 – 20
2	21 – 40
3	37 – 60
4	49 – 80
5	81 – 100

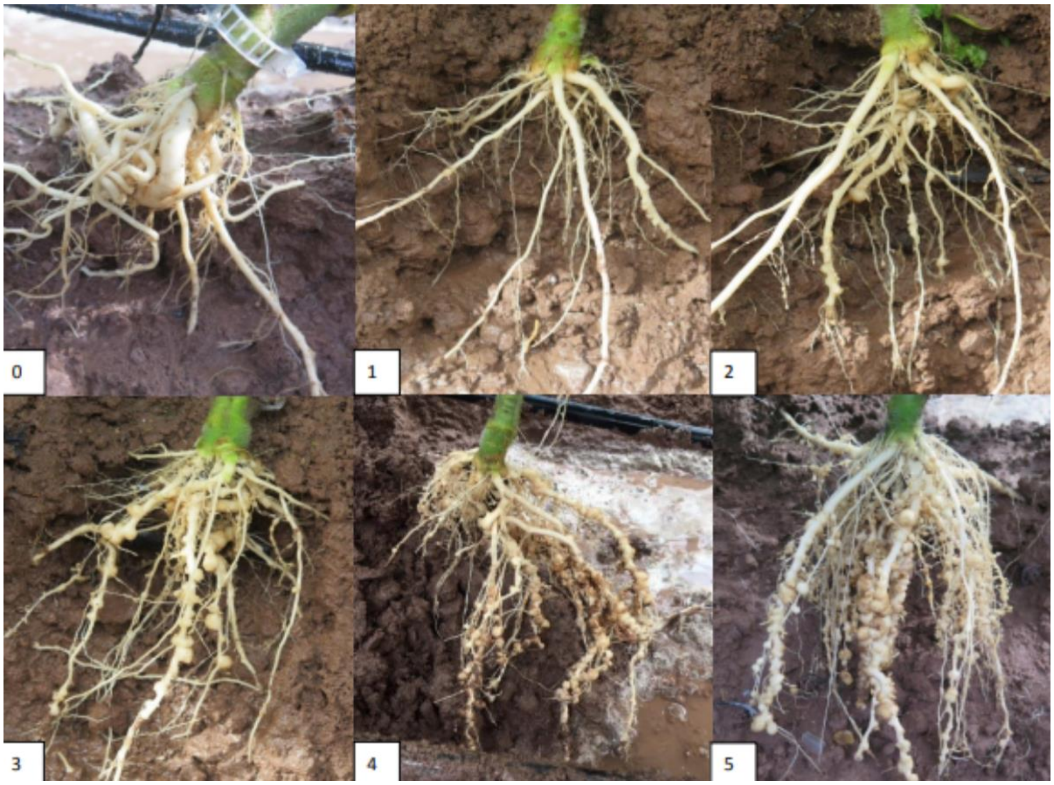

Supplementary Figure 1. Visual scale to evaluate the root-knot index caused by root-knot nematodes (Baker, 1978)

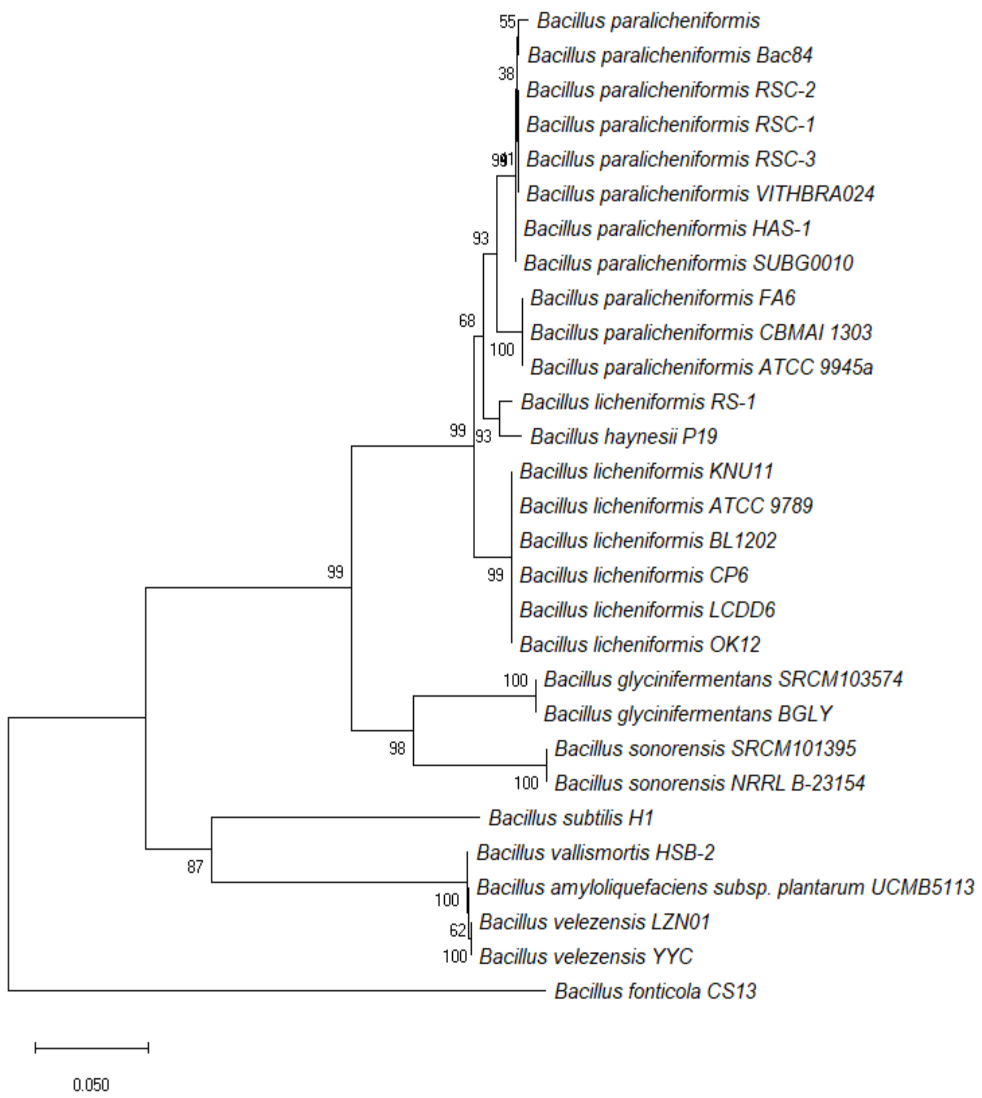

Supplementary Figure 2. Phylogenetic tree of the representative TB197 isolate and *Bacillus* spp. from NCBI GenBank based on the *gyrA* gene sequence was constructed using the neighbor-joining method with 1000 replicates. Bar indicates 0.005 substitutions per nucleotide position.

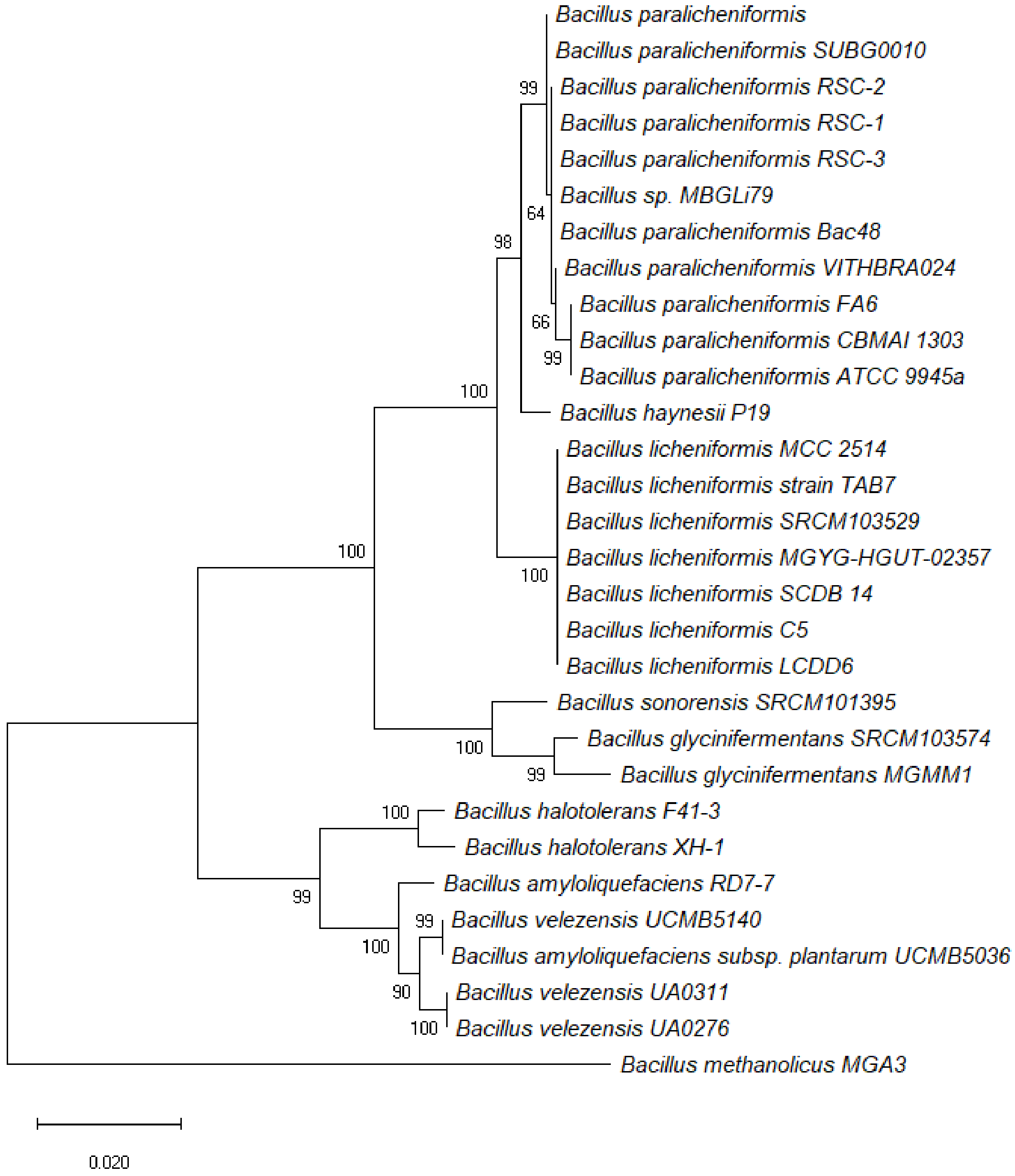

Supplementary Figure 3. Phylogenetic tree of the representative TB197 isolate and *Bacillus* spp. from NCBI GenBank based on the *groEL* gene sequence was constructed using the neighbor-joining method with 1000 replicates. Bar indicates 0.020 substitutions per nucleotide position.
